# Effect of 30 Hz theta burst transcranial magnetic stimulation on the primary motor cortex in children and adolescents

**DOI:** 10.3389/fnhum.2015.00091

**Published:** 2015-02-25

**Authors:** Ernest V. Pedapati, Donald L. Gilbert, Paul S. Horn, David A. Huddleston, Cameron S. Laue, Nasrin Shahana, Steve W. Wu

**Affiliations:** ^1^Division of Neurology, Cincinnati Children's Hospital Medical CenterCincinnati, OH, USA; ^2^Division of Child and Adolescent Psychiatry, Cincinnati Children's Hospital Medical CenterCincinnati, OH, USA; ^3^Division of Biostatistics and Epidemiology, Cincinnati Children's Hospital Medical CenterCincinnati, OH, USA

**Keywords:** repetitive transcranial magnetic stimulation, theta burst stimulation, long-term potentiation, pediatric, neuroplasticity

## Abstract

Fourteen healthy children (13.8 ± 2.2 years, range 10–16; M:F = 5:9) received 30 Hz intermittent theta burst transcranial magnetic stimulation (iTBS) with a stimulation intensity of 70% of resting motor threshold (RMT) with a total of 300 (iTBS300) pulses. All volunteers were free of neurologic, psychiatric and serious medical illnesses, not taking any neuropsychiatric medications, and did not have any contraindications to transcranial magnetic stimulation. Changes in the mean amplitudes of motor-evoked potentials from baseline following iTBS were expressed as a ratio and assessed from 1 to 10 min (BLOCK1) and 1–30 min (BLOCK2) using repeated-measures analysis of variance. All 14 subjects completed iTBS300 over the dominant primary motor cortex (M1) without any clinically reported adverse events. ITBS300 produced significant M1 facilitation [*F*_(5, 65)_ = 3.165, *p* = 0.01] at BLOCK1 and trend level M1 facilitation at BLOCK2 [*F*_(10, 129)_ = 1.69, *p* = 0.089]. Although iTBS300 (stimulation duration of 92 s at 70% RMT) delivered over M1 in typically developed children was well-tolerated and produced on average significant facilitatory changes in cortical excitability, the post-iTBS300 neurophysiologic response was variable in our small sample. ITBS300-induced changes may represent a potential neuroplastic biomarker in healthy children and those with neuro-genetic or neuro-psychiatric disorders. However, a larger sample size is needed to address safety and concerns of response variability.

## Introduction

Neuroplasticity broadly describes the ability of the nervous system to reorganize in response to intrinsic or environmental demands and underlies the conceptual framework of learning, memory and development (Lamprecht and LeDoux, 2004; Pascual-Leone et al., 2005). Though genetic and early environmental factors dictate the potential scope of brain development, neuroplastic processes play a critical role following birth to configure and optimize neural circuits, including the maturation of complex sensory, cognitive and regulatory functions throughout life (Tau and Peterson, 2009). Moreover, there is evidence that, for a broad group of neurodevelopmental disorders, abnormalities in the mechanisms of neuroplasticity, including maladaptive plasticity (Johnston, 2004), may best explain the fundamental pathophysiology of these disorders, including Fragile X Syndrome (Huber et al., 2002), Neurofibramatosis-1 (Costa et al., 2002), Gilles de la Tourette's syndrome (Wu and Gilbert, 2012), and autism spectrum disorders (Markram and Markram, 2010).

Despite relevance of aberrant neuroplasticity in animal models of multiple neurodevelopmental disorders, little is known of the role of long-term potentiation (LTP) and the relationship with behavioral plasticity in the typical developing human cortex (Martin et al., 2000). LTP describes the long-lasting modification of neuronal connections, including changes in synaptic efficacy, which is commonly cited as the cellular basis of learning and memory (Brown et al., 1988). LTP been studied extensively in mammalian hippocampus including hippocampal slices from humans undergoing temporal lobe surgery (Brown et al., 1988; Beck et al., 2000). Though investigation of cellular LTP in children have obviously been limited, electrophysiological studies of neonate and juvenile animals have shed light on the purpose and mechanisms of LTP during development. Developmental age in rodents has been associated with varying susceptibility and efficacy of induced-LTP in hippocampal slices (Harris et al., 1992; Swartzwelder et al., 1995; Leinekugel et al., 2002; Cao and Harris, 2012). In young rats, periods of susceptibility to LTP in the visual cortex coincides with developmental critical periods which can be prolonged by rearing animals in darkness (Kirkwood et al., 1995).

Transcranial magnetic stimulation (TMS) under certain stimulation parameters can lead to changes in corticospinal and corticocortical excitability that outlast the stimulation period, thus representing a surrogate marker of cellular LTP and LTD from the intact human cortex (Pascual-Leone et al., 1994). These phenomena share a remarkable similarity to cellular measurements of LTP or LTD, including the loss of TMS-induced LTP- and LTD-like effects after N-methyl-D-aspartate receptor blockade (Stefan et al., 2002; Wolters et al., 2003; Huang et al., 2007) and methods of physiological induction, either through tetanic stimulation, such as theta burst stimulation (TBS) (Pascual-Leone et al., 1994; Huang and Rothwell, 2004; Huang et al., 2005) or through associative methods, such as paired associative stimulation (PAS) (Stefan et al., 2000). Though details regarding individual synaptic connections are at best speculative, TMS techniques can grossly quantify the final output of a specific region of the neocortex and test hypotheses regarding the configuration of established neural networks.

Here we report the effect of a modified 30 Hz intermittent TBS (iTBS) protocol, previously reported to generate primary motor (M1) cortical facilitation in adults (Wu et al., 2012a) and now optimized for the pediatric population, on M1 excitability of typically-developing children/adolescents. Despite several pediatric repetitive TMS (rTMS) studies (Kirton et al., 2008; Oberman et al., 2010, 2014; Wu and Gilbert, 2012; Gillick et al., 2014; Wu et al., 2014), there is limited data on the effect of rTMS and TBS on the developing cortex. In addition, the reported inter-individual variability to iTBS potentially limits the use of the technique as a diagnostic or prognostic tool (Hamada et al., 2013; Lopez-Alonso et al., 2014). The overall goal of this work was to establish a safe biomarker of pediatric neuronal plasticity using iTBS. Such a marker would provide an additional tool to explore the cortical physiology of suspected neuroplastic abnormalities across a host of pediatric illnesses of the central nervous system. In addition, we systematically discuss the rationale for the modification of TBS parameters based on safety concerns and feasibility for use in children. The present study, to our knowledge, represents the first published cohort of healthy children who have undergone iTBS. We hypothesized that 30 Hz iTBS to M1 in healthy children would elicit a brief physiological facilitation of motor-evoked potential (MEP) amplitudes following stimulation.

## Materials and methods

Parents of pediatric patients gave written informed consent and child participants gave written informed assent for the study, which were approved by the Cincinnati Children's Hospital Medical Center Institutional Review Board. Participants were reimbursed for time and travel.

### Participants

Healthy children ages 8–17 were recruited through advertising flyers and email through the local institution and community. All volunteers were free of neurologic, psychiatric and serious medical illnesses, were not taking any neuropsychiatric medications, and did not have any contraindications to TMS (Rossi et al., 2011). Handedness was either determined through Physical And Neurological Examination for Soft Signs (Denckla, 1985) or the Edinburgh Handedness Inventory (Oldfield, 1971).

### Single pulse transcranial magnetic stimulation (spTMS)

A monophasic Magstim 200 stimulator connected to a figure-8, 70 mm coil (Magstim Ltd., Whitland, UK) was used to determine resting motor threshold (RMT) and obtain MEPs measured by surface electromyography (EMG) in the first dorsal interosseous (FDI) muscle of the dominant hand. A second set of EMG leads was placed on dominant extensor carpi radialis for monitoring during iTBS. Participants were seated comfortably with both arms fully supported on a pillow. Full muscle relaxation was monitored visually and by EMG. The figure-8 coil (handle pointing posteriorly at 45°) was placed tangentially to the scalp over the dominant M1 at the optimal site for obtaining maximal peak-to-peak amplitude of MEPs from the dominant FDI using standard methods (Mills and Nithi, 1997). This “hot spot” was marked with a wax pencil for consistent placement of the figure-8 coils during application of spTMS and rTMS. We opted not to employ neuronavigation as (1) TBS of the motor cortex has been routinely performed with a non-technical approach (Huang et al., 2005) and (2) to maximize the potential feasibility of the protocol for widespread biomarker use. TMS pulses separated by 6 s (±5%; generated by Signal software version 2.15; Cambridge Electronic Design Limited, Cambridge, UK) were administered at intensities of 1.2^*^baseline RMT to obtain MEP amplitudes at 11 time points: 20 pulses (114 s) at baseline (T0), and 10 pulses (54 s) at 1 (T1), 3 (T2), 5 (T3), 7 (T4), 10 (T5), 12.5 (T6), 15 (T7), 17.5 (T8), 20 (T9), and 30 (T10) min following iTBS. Surface EMG signals were amplified and filtered (100/1000 Hz; Coulbourn Instruments, Allentown, PA) before being digitized at 2 kHz and stored for analysis, using Signal software and a Micro1401 interface (Cambridge Electronic Design, Cambridge, UK). Each surface EMG tracing was reviewed offline and tagged for removal if it contained muscle movements prior to the TMS pulse (~1% of all tracings). Due to technical difficulties, there was missing data for the T8 time point for one subject.

### Measurement of resting motor threshold

RMT was defined for each Magstim stimulator separately as the minimal intensity of stimulation to the dominant M1 to induce MEPs in at least 3 out of 6 consecutive trials following determination of the optimal site (Conforto et al., 2004). Stimulation began well above threshold intensity, usually 75% of maximal stimulator output) and decreased until RMT was identified within a 1% increment. Due to the influence of phasic and tonic finger movements on TBS outcome (Gentner et al., 2008; Huang et al., 2008; Iezzi et al., 2008), we chose not to measure active motor threshold and instead used RMT as a reference for stimulation intensity.

### Intermittent theta burst stimulation

**Figure 1:**
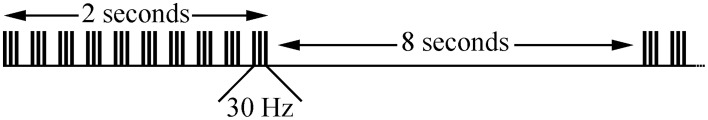
**Pulse pattern for iTBS300**. Three TMS pulses per burst were given at 30 Hz. A burst was delivered every 200 ms so that 10 bursts were given in a 2-s train. Ten trains were given every 10 s for a total of 300 magnetic pulses per iTBS300.

ITBS was performed using a biphasic 115V version of Magstim SuperRapid^2^Plus^1^ (Magstim Ltd., Whitland, UK) connected to a figure-8, 70 mm coil applied to the M1 “hot spot” as designated above. We did not use additional hearing protection as in laboratory measurement of mean (less than 57.9 dB) and peak decibel levels (less than 69.1 dB) of single pulse TMS and iTBS fell within well-established hearing safety standards and consistent with previous reports (Dhamne et al., 2014). The use of Magstim 200 in addition to the SuperRapid^2^Plus^1^ allowed for the measurement of RMT in children who generally have higher thresholds (Garvey et al., 2003). All iTBS sessions were performed in the afternoon. Subjects received iTBS300 (Figure 1), which consisted of bursts of 3 magnetic pulses at 30 Hz repeating every 200 ms for 2 s (one train) with trains repeated every 10 s apart for total of 300 pulses (92 s) at a stimulation intensity of 70%^*^RMT. Full muscle relaxation and generation of evoked potentials by iTBS was monitored visually and by continuous EMG throughout the iTBS stimulation period.

Thirty Hz iTBS was used, rather than the more typical 50 Hz, as this allows for higher stimulation intensities (i.e., 30 Hz TBS can be delivered at up to 89% power vs. only 57% for 50 Hz TBS with Magstim SuperRapid^2^Plus^1^). Moreover, 30 Hz TBS has been shown to produce the expected LTP- and LTD-like changes in M1 (Goldsworthy et al., 2012; Wu and Gilbert, 2012). Higher stimulation intensity is often necessary for TBS research in children as they have higher motor thresholds (Garvey et al., 2003). The 70%^*^RMT intensity was chosen to balance safety (the only case of TBS-induced seizure occurred at 100%^*^RMT) (Oberman and Pascual-Leone, 2009) and efficacy (i.e., we expected higher facilitatory changes in M1 excitability with higher stimulation intensity). Before and immediately after the 30-min time point, a structured diagnostic interview with detailed 16-question review of systems [headache, scalp pain, arm/hand pain, other pain(s), numbness/tingling, other sensation(s), weakness, loss of dexterity, vision/hearing change(s), ear ringing, nausea/vomiting, appetite loss, rash, skin change(s) or any other symptom(s)] was conducted to rate any potential adverse events on an ordinal scale (none, minimal, mild, moderate, marked, or severe) (Wu et al., 2012b).

### Statistical analysis

Descriptive statistics were applied to demographic and baseline physiological measures. Mean MEP fold change was normalized to be expressed as a ratio of average post-TBS/pre-TBS MEP peak-to-peak amplitudes for each time point. Since the iTBS300 protocol has half the total pulses compared to the original description, we anticipated a shorter duration of iTBS effect on cortical excitability (Huang et al., 2005). Therefore, two repeated measures analysis of variance (RM-ANOVA) were performed, analyzing MEP-fold change by a within-subject factor for 10 min—BLOCK1 (6 levels: T0; T1; T2; T3; T4; T5)—and for 30 min—BLOCK2 (11 levels: T0; T1; T2; T3; T4; T5; T6; T7; T8; T9; T10). We tested the hypotheses that the modified iTBS protocol would produce facilitation of mean MEP fold-change across BLOCK1 and BLOCK2. All analyses were performed in SAS (SAS Institute Inc., Cary, NC, USA) with a two-tailed *p* < 0.05 considered significant. To determine whether age had an effect on the post-iTBS300 change, it was included as a covariate in the RM-ANOVA.

In addition to RM-ANOVA, which has been used in most prior published TBS studies, we performed a secondary linear mixed model (LMM) analysis which has several potential advantages. This analysis incorporates intrasubject correlations, accounts well for missing observations, and, by using raw MEPs, accounts for inter-individual variability in the baseline MEP amplitudes (Huang et al., 2005; Wu et al., 2012a; Dhamne et al., 2014). This is a special case of a linear mixed model (LMM) with the added component of a within subject covariance structure to account for the repeated measures over time. We used an unstructured covariance model in which the correlation between any two values within subject is estimated from the data (West et al., 2006). Our a priori hypothesis expected the adjusted mean amplitudes at each post-TBS time point differed from baseline. For each comparison, the resultant *p*-value were corrected for a False Discovery Rate (FDR) to account for the multiple testing (Benjamini and Hochberg, 1995); with 5 and 10 contrasts, respectively, for the two blocks.

## Results

### Demographics and safety

Fourteen healthy children (13.8 ± 2.2 years, range 10–16; M:F = 5:9) completed the study (Table 1). Thirteen subjects were right-handed. No adverse events were reported or identified by structured diagnostic interviews and no seizure occurred.

**Table 1:** **Effect of modified iTBS300 on M1 as measured by MEP-fold change from baseline over time in healthy children (*n* = 14, 13.8 ± 2.2 years)**.

**Minutes after iTBS300**	**1**	**3**	**5**	**7**	**10**	**12.5**	**15**	**17.5**	**20**	**30**
Mean MEP fold change	1.40	1.45	1.30	1.20	1.13	1.23	1.32	1.51	1.55	1.25
SD	0.36	0.45	0.51	0.47	0.47	0.67	0.59	1.08	0.98	0.56
SEM	0.10	0.12	0.14	0.12	0.12	0.18	0.16	0.30	0.26	0.15
N	14	14	14	14	14	14	14	13	14	14
RM-ANOVA	BLOCK1, *F*_(5, 65)_ = 3.17, *p* = 0.01^*^									
	BLOCK2, *F*_(10, 129)_ = 1.69, *p* = 0.089									
LMM	BLOCK1, *F*_(5, 13)_ = 4.72, *p* = 0.01^*^									
	BLOCK2, *F*_(10, 13)_ = 6.28, *p* = 0.002^*^									

BLOCK1, analysis of 1–10 min; BLOCK2, analysis of 1–30 min. SD, standard deviation; SEM, standard error of the mean; n, number of observations; MEP, Motor-Evoked Potentials; iTBS, intermittent Theta Burst Stimulation; M1, primary motor cortex; RM-ANOVA, repeated measures analysis of variance; LMM, Linear Mixed Model;

* *indicates statistical significance*.

### iTBS 300

Average RMT was 50.7 ± 9.7% of Magstim200 maximal output and 63.7 ± 13.6% of SuperRapid^2^Plus^1^ maximal output. The “hot-spot” scalp location was identical for both machines. Mean iTBS stimulation intensity was 44.6 ± 9.5% (range: 31–62%) of SuperRapid^2^Plus^1^ maximal output.

**Figure 2:**
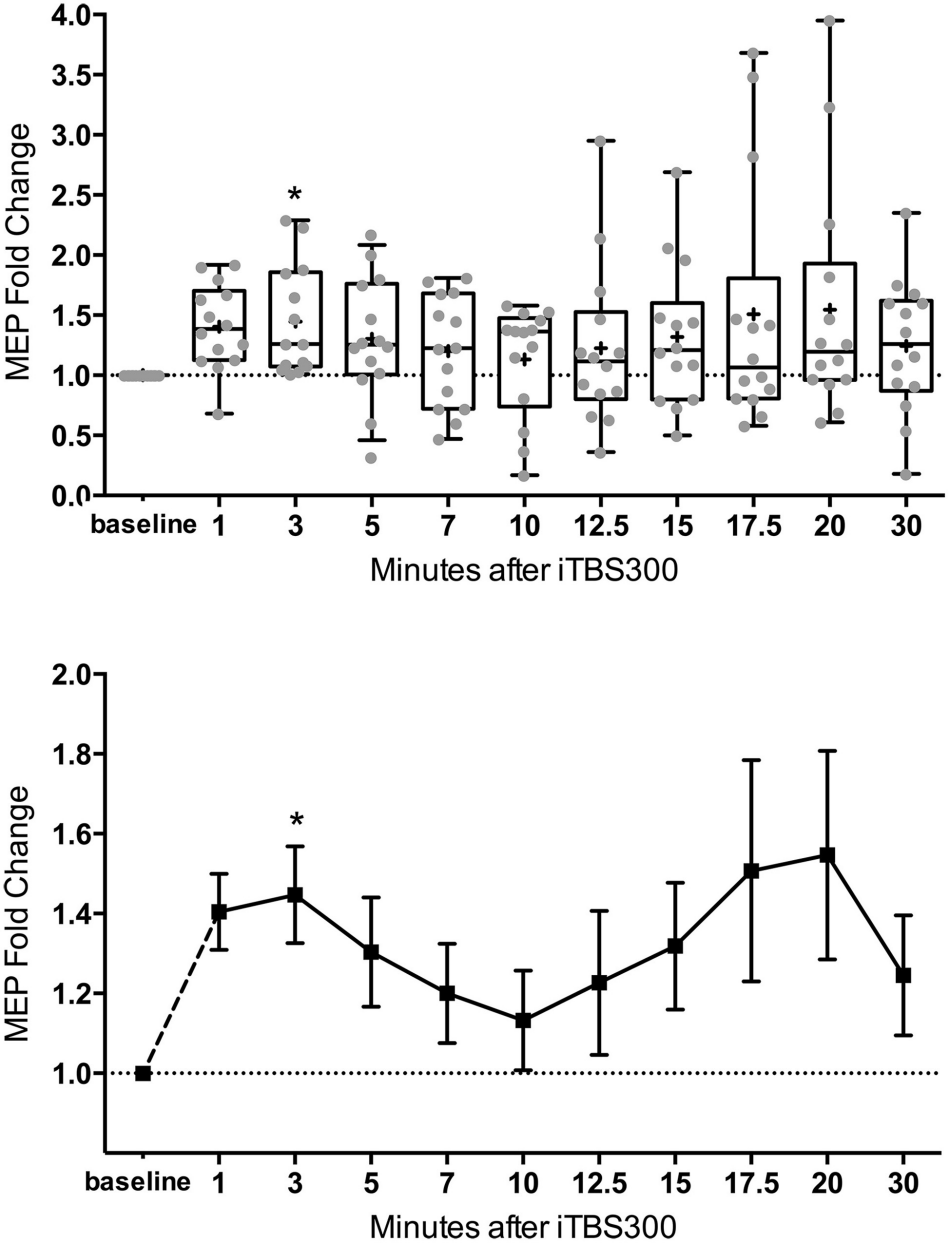
**M1 excitability changes after 30 Hz iTBS (300 pulses, 70% of RMT) in healthy children (*n* = 14, mean age = 13.8 ± 2.2 years old) from 1 to 30 min. (Top)** Box (25th–75th) and Whiskers (5th–95th) percentiles with line at median and cross at mean. Mean MEP-fold change of each subject is overlaid as gray circles. **(Bottom)** Mean line plot of MEP-fold changes with error bars representing standard error of means. MEP, Motor-Evoked Potentials; RMT, resting motor threshold; iTBS, intermittent Theta Burst Stimulation; M1, primary motor cortex; * denotes statistically significant increase compared to baseline in *post-hoc* analysis after correcting for multiple comparison.

The mean MEP fold changes for each time point and RM-ANOVA and LMM results following iTBS are summarized in Table 1. One-Way RM-ANOVA revealed a significant facilitation during BLOCK1 (1–10 min) and trend level facilitation during BLOCK2 (1–30 min) (Figure 2). For LMM, the main effect Time was statistically significant for both blocks. For BLOCK1, after adjusting for multiple comparisons, the MEP-amplitudes at 3 min were significantly larger than baseline (FDR adjusted *p* = 0.021). For BLOCK2, after adjusting for multiple comparisons, the MEP amplitudes at 3 min were significantly larger than baseline (FDR adjusted *p* = 0.042). Adding age as a covariate did not have a significant effect in either analysis (not shown).

## Discussion

In the present study, we demonstrated that a 300 pulse, intermittent theta burst stimulation (iTBS) protocol delivered at sub-motor threshold intensity resulted in facilitation of M1 cortical excitability in healthy children. Nearly all prior studies have been performed in adults (Oberman et al., 2011; Wu et al., 2012a). In place of the originally described 50 Hz bursts (Huang et al., 2005), we used 30 Hz TBS to create a frequency/intensity paradigm that was compatible with the mechanical parameters of the SuperRapid^2^Plus^1^ device for use in pediatric research (Wu et al., 2012a). Using the commonly employed method of analysis (repeated-measures ANOVA) in rTMS studies, the iTBS300 protocol demonstrated a statistically significant M1 facilitation from 1 to 10 min. However, there was significant variability in M1 response. *Post-hoc* analysis using an alternative analytical technique (LMM) and correcting for multiple comparisons showed that this facilitatory effect on M1 was primarily seen at the 3-min time point following iTBS300. Even with these limitations, the main conclusion of this study is that we were able to safely deliver iTBS to typically developing children with statistically significant facilitatory changes in M1 excitability thus lending support for further judicious use of iTBS to understand neuroplasticity in the developing cortex in children with developmental disorders.

### iTBS-induced facilitation in M1 excitability

To our knowledge, this is the first report of iTBS induced M1 neurophysiologic changes in healthy children. These results demonstrate similar magnitude of facilitation as we found in adults using 30 Hz iTBS with 600 pulses at 90% of RMT (Wu et al., 2012a). A brief, non-invasive method of inducing LTP- and LTD-like changes in cortical excitability holds tremendous potential to advance the study of neurodevelopmental processes (Morris et al., 2014). The optimization and validation of these techniques can also provide insight into the neural mechanisms of learning and rehabilitation (Johnston, 2009) and bridge decades of electrophysiological research from *in vivo* and *in vitro* models of central nervous system disease with clinically recognized motor, cognitive, or emotional impairments in humans (Freitas et al., 2011; Castren et al., 2012). To date, the most commonly used TMS techniques to induce cortical excitability changes are paired associative stimulation (PAS), rTMS, or TBS (Stefan et al., 2000; Di Lazzaro et al., 2011). In sensitive populations, rTMS and PAS may be limited by the discomfort of prolonged periods of stimulation above motor threshold.

Virtually all other TBS studies of M1 plasticity in adults have been performed using 50 Hz stimulation with relatively lower stimulation intensities (Huang et al., 2005; Cardenas-Morales et al., 2010; Hoogendam et al., 2010). Although there are several similarities in our results, including the time course and maximal changes in MEP amplitudes (Huang et al., 2005; Wu et al., 2012a; Cardenas-Morales et al., 2014), comparing effects in adults vs. children and 50 Hz vs. 30 Hz iTBS will require further study.

Of interest and relevance to future studies, we also confirmed the statistical significance of the facilitation effect over 10 min (BLOCK1) using a linear mixed model (LMM) analysis. Using LMM, the BLOCK2 time effect was also statistically significant, whereas for the more commonly used RM-ANOVA found significance at the trend level. A repeated-measures LMM has several advantages over a traditional multivariate approach where there is no ability to model the correlation between observations on the same subject (Krueger and Tian, 2004). An LMM allows the incorporation of intrasubject correlations and since each observation is considered individually (including continuous or categorical covariates at a particular time) this model can also account for missing observations without list-wise deletion. We created an LMM model that used an unstructured covariance for the raw MEP amplitudes that closely fit our data. By using the raw MEPs, we were able to maintain the variability of individual subjects baseline, which is lost when looking at a MEP-fold change and transforming the baseline to 1. This may account for the discrepancy between the multivariate approach and the LMM results for BLOCK2. *Post-hoc* testing against this baseline in both time blocks, including a stringent correction for multiple comparisons, found a significant contrast at 3 min. Thus, both performed analyses support a statistically significant facilitating effect on cortical excitability within the first 10 min.

### Variability in iTBS-induced facilitation in M1 excitability

One significant concern about TBS induced plasticity is the variability of the magnitude and direction of the MEP response (Player et al., 2012; Hamada et al., 2013; Hinder et al., 2014). In adults, several factors have been identified to contribute to this variability including age, gender, time of day, genetic background, and attention (Cardenas-Morales et al., 2010; Hoogendam et al., 2010; Ridding and Ziemann, 2010). Furthermore, intrinsic mechanisms such as inter-individual differences in the recruitment of interneuron networks by TMS may play a larger role than previously realized (Hamada et al., 2013). Although covariate analysis of our data did not find an effect of age, this and other factors should be analyzed in future, larger studies. Over a 30 min time course BLOCK2, as has also been reported after conventional TBS (Huang et al., 2005), we observed a second “peak” (see Table 1 and Figure 2). In cellular models, LTP has come to be recognized as a phenomenon that represents a series of phases, including early and late, that can be more precisely categorized based on molecular mechanisms and order of persistence (Raymond, 2007).

### Metaplasticity

The concept of the previous brain activity affecting synaptic response is termed metaplasticity. Several studies have shown that tonic or phasic finger movements before TBS can change the expected outcome of the tetanic stimulation (Gentner et al., 2008; Huang et al., 2008; Iezzi et al., 2008). Prior brain stimulation may also “prime” TBS response (Todd and Ridding, 2010), such as prolonging the duration of the stimulation (i.e., more pulses). In addition, extending the number of pulses seems to influence the results. One study found that facilitation and inhibition could be reversed simply by doubling the TBS pulses delivered from 600 to 1200 (iTBS1200) (Gamboa et al., 2010), while another reported that 1800 pulses of iTBS resulted in significantly higher facilitation of MEP-amplitudes than iTBS600 or iTBS1200 (Nettekoven et al., 2014). Given concerns for feasibility in pediatric populations, we were interested to study iTBS with fewer pulses. The iTBS300 protocol presented in this study produced an increase in M1 excitability in most pediatric subjects. So far, there has been one iTBS150 study that showed no significant M1 changes in adults (Huang et al., 2008). Future studies in children could evaluate iTBS150 to determine if this is sufficient to modulate cortical excitability.

It is possible that the 0.17 Hz test pulses used prior to (baseline) and after iTBS may themselves induce metaplastic effects as was suggested by a recent study of PAS-induced LTP and LTD (Delvendahl et al., 2010). However, the 0.1 Hz rTMS precondition in this study abolished PAS-induced neuroplastic effects whereas we observed statistically significant increase in M1 excitation in our study. Furthermore, very low frequency rTMS (0.1 and 0.2 Hz) have not been shown to exert direct effect on MEP amplitudes (Chen et al., 1997; Delvendahl et al., 2010; Furukawa et al., 2010). This could create a trade-off between using more frequent TMS to capture the temporal characteristics of induced cortical excitability vs. less frequent TMS to avoid inducing metaplastic effects. Future sham controlled TBS studies (Davis et al., 2013), or perhaps studies outside of motor cortex with different outputs, may clarify this.

### Safety

A key finding of this study is that iTBS was delivered safely and without any reported clinical adverse effects in all 14 children who participated in this study. This is an important finding as there is limited data on the use of TBS in the pediatric population (Oberman et al., 2010, 2014; Wu et al., 2014; Hong et al., 2015). A small number of these participants reported mild adverse events after TBS: fatigue, headache/scalp pain, arm/hand pain, paresthesia, weakness, nausea, tinnitus, abdominal pain and dry eyes. We recently compared the adverse event rates between TBS and single-/paired-pulse TMS sessions in 165 children and found no significant difference (Hong et al., 2015). In the present study, systematic review of systems following iTBS found no significant adverse effects. There are a few possible explanations for this. First, the iTBS protocol contained only 300 pulses rather than the originally described 600 pulses. Thus, this 92-s TBS stimulation duration may have a lower probably of causing adverse effects. Second, based on a systematic review of >1000 adults who received >4500 TBS sessions, a crude risk of 1.1% was identified for mild adverse events (Oberman et al., 2011). Furthermore, another safety report of various forms of TMS/rTMS in 113 adults showed that TBS sessions were associated with less adverse events (Maizey et al., 2013). These adult safety data may also explain why we did not detect any adverse events in our small pediatric sample.

### Limitations

The results of this study are vulnerable to a type II error given the small sample size. Thus, we could not adequately examine factors that might lead to variability in iTBS300 response. Generalization of our findings may be limited by the predominance of females in our cohort, as gender difference may be a determinant of TMS-induced plasticity (Ridding and Ziemann, 2010).

In addition, further work needs to be done to extend these types of assessments to younger children. Although we attempted to recruit younger children for the study, several participants' motor thresholds were too high to proceed with TBS. The youngest subject in our cohort was 9 years old but his RMT was relatively low for his age (54% on Magstim200, 68% on SuperRapid^2^Plus^1^) which allowed us to complete the iTBS protocol. In rodent models, the maturation of the cortex with advancing developmental age influences the conditions necessary to induce LTP-effects (Meredith et al., 2003). Such an analysis lies outside the scope of the present study, however, with a larger sample with younger age groups may allow the quantification of such effects in the future. In addition, repeated sessions could be used to evaluate the extent of intra-individual variability, for example, related to time of day, stress, fatigue, or hormonal fluctuations in females.

### Conclusion

This is the first report of iTBS- induced M1 neurophysiologic effects in healthy children. All participants safely completed the iTBS300 session which involved just 92 s of bursts of subthreshold TMS pulses without any serious adverse events. We were able to show statistically significant increase in M1 excitability in the first 10 min after iTBS300. Future pediatric TBS studies to acquire normative data are needed. We speculate the demonstrated physiological effects of this protocol to M1 could also be further investigated in non-motor regions for neuromodulation or for repeated applications in clinical trials. This data supports further, judicious use of iTBS as a technique for studying brain development, neuropsychiatric and neuro-developmental disorders.

### Conflict of interest statement

The authors declare that the research was conducted in the absence of any commercial or financial relationships that could be construed as a potential conflict of interest.

## References

[B1] Beck H. Goussakov I. V. Lie A. Helmstaedter C. Elger C. E. (2000). Synaptic plasticity in the human dentate gyrus. J. Neurosci. 20, 7080–7086. 10995855 10.1523/JNEUROSCI.20-18-07080.2000 PMC6772802

[B2] Benjamini Y. Hochberg Y. (1995). Controlling the false discovery rate: a practical and powerful approach to multiple testing. J. R. Stat. Soc. B 57, 289–300.

[B3] Brown T. H. Chapman P. F. Kairiss E. W. Keenan C. L. (1988). Long-term synaptic potentiation. Science 242, 724–728. 10.1126/science.2903551 2903551

[B4] Cao G. Harris K. M. (2012). Developmental regulation of the late phase of long-term potentiation (L-LTP) and metaplasticity in hippocampal area CA1 of the rat. J. Neurophysiol. 107, 902–912. 10.1152/jn.00780.2011 22114158 PMC3289468

[B5] Cardenas-Morales L. Nowak D. A. Kammer T. Wolf R. C. Schonfeldt-Lecuona C. (2010). Mechanisms and applications of theta-burst rTMS on the human motor cortex. Brain Topogr. 22, 294–306. 10.1007/s10548-009-0084-7 19288184

[B6] Cardenas-Morales L. Volz L. J. Michely J. Rehme A. K. Pool E. M. Nettekoven C.. (2014). Network connectivity and individual responses to brain stimulation in the human motor system. Cereb. Cortex 24, 1697–1707. 10.1093/cercor/bht023 23395849

[B7] Castren E. Elgersma Y. Maffei L. Hagerman R. (2012). Treatment of neurodevelopmental disorders in adulthood. J. Neurosci. 32, 14074–14079. 10.1523/JNEUROSCI.3287-12.2012 23055475 PMC3500763

[B8] Chen R. Classen J. Gerloff C. Celnik P. Wassermann E. M. Hallett M.. (1997). Depression of motor cortex excitability by low-frequency transcranial magnetic stimulation. Neurology 48, 1398–1403. 10.1212/WNL.48.5.1398 9153480

[B9] Conforto A. B. Z'Graggen W. J. Kohl A. S. Rosler K. M. Kaelin-Lang A. (2004). Impact of coil position and electrophysiological monitoring on determination of motor thresholds to transcranial magnetic stimulation. Clin. Neurophysiol. 115, 812–819. 10.1016/j.clinph.2003.11.010 15003761

[B10] Costa R. M. Federov N. B. Kogan J. H. Murphy G. G. Stern J. Ohno M.. (2002). Mechanism for the learning deficits in a mouse model of neurofibromatosis type 1. Nature 415, 526–530. 10.1038/nature711 11793011

[B11] Davis N. J. Gold E. Pascual-Leone A. Bracewell R. M. (2013). Challenges of proper placebo control for non-invasive brain stimulation in clinical and experimental applications. Eur. J. Neurosci. [Epub ahead of print]. 10.1111/ejn.12307 23869660

[B12] Delvendahl I. Jung N. H. Mainberger F. Kuhnke N. G. Cronjaeger M. Mall V. (2010). Occlusion of bidirectional plasticity by preceding low-frequency stimulation in the human motor cortex. Clin. Neurophysiol. 121, 594–602. 10.1016/j.clinph.2009.09.034 20074998

[B13] Denckla M. B. (1985). Revised Neurological Examination for Subtle Signs. Psychopharmacol. Bull. 21, 773–800. 4089106

[B14] Dhamne S. C. Kothare R. S. Yu C. Hsieh T. H. Anastasio E. M. Oberman L.. (2014). A measure of acoustic noise generated from transcranial magnetic stimulation coils. Brain Stimul. 7, 432–434. 10.1016/j.brs.2014.01.056 24582370

[B15] Di Lazzaro V. Dileone M. Pilato F. Capone F. Musumeci G. Ranieri F.. (2011). Modulation of motor cortex neuronal networks by rTMS: comparison of local and remote effects of six different protocols of stimulation. J. Neurophysiol. 105, 2150–2156. 10.1152/jn.00781.2010 21346213

[B16] Freitas C. Perez J. Knobel M. Tormos J. M. Oberman L. Eldaief M.. (2011). Changes in cortical plasticity across the lifespan. Front. Aging Neurosci. 3:5. 10.3389/fnagi.2011.00005 21519394 PMC3079175

[B17] Furukawa T. Toyokura M. Masakado Y. (2010). Suprathreshold 0.2 Hz repetitive transcranial magnetic stimulation (rTMS) over the prefrontal area. Tokai J. Exp. Clin. Med. 35, 29–33. 21319023

[B18] Gamboa O. L. Antal A. Moliadze V. Paulus W. (2010). Simply longer is not better: reversal of theta burst after-effect with prolonged stimulation. Exp. Brain Res. 204, 181–187 10.1007/s00221-010-2293-4 20567808 PMC2892066

[B19] Garvey M. A. Ziemann U. Bartko J. J. Denckla M. B. Barker C. A. Wassermann E. M. (2003). Cortical correlates of neuromotor development in healthy children. Clin. Neurophysiol. 114, 1662–1670. 10.1016/S1388-2457(03)00130-5 12948795

[B20] Gentner R. Wankerl K. Reinsberger C. Zeller D. Classen J. (2008). Depression of human corticospinal excitability induced by magnetic theta-burst stimulation: evidence of rapid polarity-reversing metaplasticity. Cereb. Cortex 18, 2046–2053. 10.1093/cercor/bhm239 18165282

[B21] Gillick B. T. Krach L. E. Feyma T. Rich T. L. Moberg K. Thomas W.. (2014). Primed low-frequency repetitive transcranial magnetic stimulation and constraint-induced movement therapy in pediatric hemiparesis: a randomized controlled trial. Dev. Med. Child Neurol. 56, 44–52. 10.1111/dmcn.12243 23962321 PMC3864983

[B22] Goldsworthy M. R. Pitcher J. B. Ridding M. C. (2012). A comparison of two different continuous theta burst stimulation paradigms applied to the human primary motor cortex. Clin. Neurophysiol. 123, 2256–2263. 10.1016/j.clinph.2012.05.001 22633917

[B23] Hamada M. Murase N. Hasan A. Balaratnam M. Rothwell J. C. (2013). The role of interneuron networks in driving human motor cortical plasticity. Cereb. Cortex 23, 1593–1605. 10.1093/cercor/bhs147 22661405

[B24] Harris K. M. Jensen F. E. Tsao B. (1992). Three-dimensional structure of dendritic spines and synapses in rat hippocampus (CA1) at postnatal day 15 and adult ages: implications for the maturation of synaptic physiology and long-term potentiation. J. Neurosci. 12, 2685–2705. 1613552 10.1523/JNEUROSCI.12-07-02685.1992 PMC6575840

[B25] Hinder M. R. Goss E. L. Fujiyama H. Canty A. J. Garry M. I. Rodger J.. (2014). Inter- and Intra-individual variability following intermittent theta burst stimulation: implications for rehabilitation and recovery. Brain Stimul. 7, 365–371. 10.1016/j.brs.2014.01.004 24507574

[B26] Hong J. H. Wu S. W. Pedapati E. V. Horn P. S. Huddleston D. A. Laue C. S. (2015). Safety and tolerability of theta burst stimulation versus single and paired pulse transcranial magnetic stimulation: a comparative study of 165 pediatric subjects. Front. Hum. Neurosci. 9:29 10.3389/fnhum.2015.00029 PMC4316715 25698958

[B27] Hoogendam J. M. Ramakers G. M. Di Lazzaro V. (2010). Physiology of repetitive transcranial magnetic stimulation of the human brain. Brain Stimul. 3, 95–118. 10.1016/j.brs.2009.10.005 20633438

[B28] Huang Y. Z. Rothwell J. C. (2004). The effect of short-duration bursts of high-frequency, low-intensity transcranial magnetic stimulation on the human motor cortex. Clin. Neurophysiol. 115, 1069–1075. 10.1016/j.clinph.2003.12.026 15066532

[B29] Huang Y. Z. Chen R. S. Rothwell J. C. Wen H. Y. (2007). The after-effect of human theta burst stimulation is NMDA receptor dependent. Clin. Neurophysiol. 118, 1028–1032. 10.1016/j.clinph.2007.01.021 17368094

[B30] Huang Y. Z. Edwards M. J. Rounis E. Bhatia K. P. Rothwell J. C. (2005). Theta burst stimulation of the human motor cortex. Neuron 45, 201–206. 10.1016/j.neuron.2004.12.033 15664172

[B31] Huang Y. Z. Rothwell J. C. Edwards M. J. Chen R. S. (2008). Effect of physiological activity on an NMDA-dependent form of cortical plasticity in human. Cereb. Cortex 18, 563–570. 10.1093/cercor/bhm087 17573373

[B32] Huber K. M. Gallagher S. M. Warren S. T. Bear M. F. (2002). Altered synaptic plasticity in a mouse model of fragile X mental retardation. Proc. Natl. Acad. Sci. U.S.A. 99, 7746–7750. 10.1073/pnas.122205699 12032354 PMC124340

[B33] Iezzi E. Conte A. Suppa A. Agostino R. Dinapoli L. Scontrini A.. (2008). Phasic voluntary movements reverse the aftereffects of subsequent theta-burst stimulation in humans. J. Neurophysiol. 100, 2070–2076. 10.1152/jn.90521.2008 18753328

[B34] Johnston M. V. (2004). Clinical disorders of brain plasticity. Brain Dev. 26, 73–80. 10.1016/S0387-7604(03)00102-5 15036425

[B35] Johnston M. V. (2009). Plasticity in the developing brain: implications for rehabilitation. Dev. Disabil. Res. Rev. 15, 94–101. 10.1002/ddrr.64 19489084

[B36] Kirkwood A. Lee H.-K. Bear M. F. (1995). Co-regulation of long-term potentiation and experience-dependent synaptic plasticity in visual cortex by age and experience. Nature 375, 328–331. 10.1038/375328a0 7753198

[B37] Kirton A. Chen R. Friefeld S. Gunraj C. Pontigon A. M. Deveber G. (2008). Contralesional repetitive transcranial magnetic stimulation for chronic hemiparesis in subcortical paediatric stroke: a randomised trial. Lancet Neurol. 7, 507–513. 10.1016/S1474-4422(08)70096-6 18455961

[B38] Krueger C. Tian L. (2004). A comparison of the general linear mixed model and repeated measures ANOVA using a dataset with multiple missing data points. Biol. Res. Nurs. 6, 151–157. 10.1177/1099800404267682 15388912

[B39] Lamprecht R. LeDoux J. (2004). Structural plasticity and memory. Nat. Rev. Neurosci. 5, 45–54. 10.1038/nrn1301 14708003

[B40] Leinekugel X. Khazipov R. Cannon R. Hirase H. Ben-Ari Y. Buzsaki G. (2002). Correlated bursts of activity in the neonatal hippocampus *in vivo*. Science 296, 2049–2052. 10.1126/science.1071111 12065842

[B41] Lopez-Alonso V. Cheeran B. Rio-Rodriguez D. Fernandez-Del-Olmo M. (2014). Inter-individual variability in response to non-invasive brain stimulation paradigms. Brain Stimul. 7, 372–380. 10.1016/j.brs.2014.02.004 24630849

[B42] Maizey L. Allen C. P. Dervinis M. Verbruggen F. Varnava A. Kozlov M.. (2013). Comparative incidence rates of mild adverse effects to transcranial magnetic stimulation. Clin. Neurophysiol. 124, 536–544. 10.1016/j.clinph.2012.07.024 22986284

[B43] Markram K. Markram H. (2010). The intense world theory - a unifying theory of the neurobiology of autism. Front. Hum. Neurosci. 4:224 10.3389/fnhum.2010.00224 PMC3010743 21191475

[B44] Martin S. Grimwood P. Morris R. (2000). Synaptic plasticity and memory: an evaluation of the hypothesis. Annu. Rev. Neurosci. 23, 649–711. 10.1146/annurev.neuro.23.1.649 10845078

[B45] Meredith R. M. Floyer-Lea A. M. Paulsen O. (2003). Maturation of long-term potentiation induction rules in rodent hippocampus: role of GABAergic inhibition. J. Neurosci. 23, 11142–11146. 14657173 10.1523/JNEUROSCI.23-35-11142.2003 PMC6741060

[B46] Mills K. R. Nithi K. A. (1997). Corticomotor threshold to magnetic stimulation: normal values and repeatability. Muscle Nerve 20, 570–576. 9140363 10.1002/(sici)1097-4598(199705)20:5<570::aid-mus5>3.0.co;2-6

[B47] Morris S. E. Rumsey J. M. Cuthbert B. N. (2014). Rethinking mental disorders: the role of learning and brain plasticity. Restor. Neurol. Neurosci. 32, 5–23. 10.3233/rnn-139015 23902986

[B48] Nettekoven C. Volz L. J. Kutscha M. Pool E. M. Rehme A. K. Eickhoff S. B.. (2014). Dose-dependent effects of theta burst rTMS on cortical excitability and resting-state connectivity of the human motor system. J. Neurosci. 34, 6849–6859. 10.1523/JNEUROSCI.4993-13.2014 24828639 PMC4019799

[B49] Oberman L. M. Pascual-Leone A. (2009). Report of seizure induced by continuous theta burst stimulation. Brain Stimul. 2, 246–247. 10.1016/j.brs.2009.03.003 20160904 PMC2769021

[B50] Oberman L. M. Pascual-Leone A. Rotenberg A. (2014). Modulation of corticospinal excitability by transcranial magnetic stimulation in children and adolescents with autism spectrum disorder. Front. Hum. Neurosci. 8:627. 10.3389/fnhum.2014.00627 25165441 PMC4131188

[B51] Oberman L. Edwards D. Eldaief M. Pascual-Leone A. (2011). Safety of theta burst transcranial magnetic stimulation: a systematic review of the literature. J. Clin. Neurophysiol. 28, 67–74. 10.1097/WNP.0b013e318205135f 21221011 PMC3260517

[B52] Oberman L. Ifert-Miller F. Najib U. Bashir S. Woollacott I. Gonzalez-Heydrich J. (2010). Transcranial magnetic stimulation provides means to assess cortical plasticity and excitability in humans with fragile x syndrome and autism spectrum disorder. Front. Synaptic Neurosci. 2:26 10.3389/fnsyn.2010.00026 PMC3059673 21423512

[B53] Oldfield R. C. (1971). The assessment and analysis of handedness: the Edinburgh inventory. Neuropsychologia 9, 97–113. 10.1016/0028-3932(71)90067-4 5146491

[B54] Pascual-Leone A. Amedi A. Fregni F. Merabet L. B. (2005). The plastic human brain cortex. Annu. Rev. Neurosci. 28, 377–401. 10.1146/annurev.neuro.27.070203.144216 16022601

[B55] Pascual-Leone A. Valls-Sole J. Wassermann E. M. Hallett M. (1994). Responses to rapid-rate transcranial magnetic stimulation of the human motor cortex. Brain 117(Pt 4), 847–858. 10.1093/brain/117.4.847 7922470

[B56] Player M. J. Taylor J. L. Alonzo A. Loo C. K. (2012). Paired associative stimulation increases motor cortex excitability more effectively than theta-burst stimulation. Clin. Neurophysiol. 123, 2220–2226. 10.1016/j.clinph.2012.03.081 22608487

[B57] Raymond C. R. (2007). LTP forms 1, 2 and 3: different mechanisms for the “long” in long-term potentiation. Trends Neurosci. 30, 167–175. 10.1016/j.tins.2007.01.007 17292975

[B58] Ridding M. C. Ziemann U. (2010). Determinants of the induction of cortical plasticity by non-invasive brain stimulation in healthy subjects. J. Physiol. 588, 2291–2304. 10.1113/jphysiol.2010.190314 20478978 PMC2915507

[B59] Rossi S. Hallett M. Rossini P. M. Pascual-Leone A. (2011). Screening questionnaire before TMS: an update. Clin. Neurophysiol. 122, 1686. 10.1016/j.clinph.2010.12.037 21227747

[B60] Stefan K. Kunesch E. Benecke R. Cohen L. G. Classen J. (2002). Mechanisms of enhancement of human motor cortex excitability induced by interventional paired associative stimulation. J. Physiol. 543(Pt 2), 699–708 10.1113/jphysiol.2002.023317 12205201 PMC2290505

[B61] Stefan K. Kunesch E. Cohen L. G. Benecke R. Classen J. (2000). Induction of plasticity in the human motor cortex by paired associative stimulation. Brain 123, 572–584. 10.1093/brain/123.3.572 10686179

[B62] Swartzwelder H. S. Wilson W. Tayyeb M. (1995). Age-dependent inhibition of long-term potentiation by ethanol in immature versus mature Hippocampus. Alcohol. Clin. Exp. Res. 19, 1480–1485. 10.1111/j.1530-0277.1995.tb01011.x 8749814

[B63] Tau G. Z. Peterson B. S. (2009). Normal development of brain circuits. Neuropsychopharmacology 35, 147–168 10.1038/npp.2009.115 19794405 PMC3055433

[B64] Todd G. Ridding M. C. (2010). The response to repetitive stimulation of human motor cortex is influenced by the history of synaptic activity. Restor. Neurol. Neurosci. 28, 459–467. 10.3233/RNN-2010-0565 20714070

[B65] West B. T. Welch K. B. Galecki A. T. (2006). Linear Mixed Models: a Practical Guide Using Statistical Software. Boca Raton, FL: CRC Press.

[B66] Wolters A. Sandbrink F. Schlottmann A. Kunesch E. Stefan K. Cohen L. G.. (2003). A temporally asymmetric Hebbian rule governing plasticity in the human motor cortex. J. Neurophysiol. 89, 2339–2345. 10.1152/jn.00900.2002 12612033

[B67] Wu S. W. Gilbert D. L. (2012). Altered neurophysiologic response to intermittent theta burst stimulation in Tourette syndrome. Brain Stimul. 5, 315–319. 10.1016/j.brs.2011.04.001 22037119

[B68] Wu S. W. Maloney T. Gilbert D. L. Dixon S. G. Horn P. S. Huddleston D. A.. (2014). Functional MRI-navigated repetitive transcranial magnetic stimulation over supplementary motor area in chronic tic disorders. Brain Stimul. 7, 212–218. 10.1016/j.brs.2013.10.005 24268723

[B69] Wu S. W. Shahana N. Huddleston D. A. Gilbert D. L. (2012a). Effects of 30Hz theta burst transcranial magnetic stimulation on the primary motor cortex. J. Neurosci. Methods 208, 161–164. 10.1016/j.jneumeth.2012.05.014 22627376 PMC3398243

[B70] Wu S. W. Shahana N. Huddleston D. A. Lewis A. N. Gilbert D. L. (2012b). Safety and tolerability of theta-burst transcranial magnetic stimulation in children. Dev. Med. Child Neurol. 54, 636–639. 10.1111/j.1469-8749.2012.04300.x 22515662

